# The effects of Kanizsa contours on temporal integration and attention in rapid serial visual presentation

**DOI:** 10.3758/s13414-017-1333-6

**Published:** 2017-05-19

**Authors:** Aytaç Karabay, Elkan G. Akyürek

**Affiliations:** Department of Psychology, Experimental Psychology, University of Groningen, Grote Kruisstraat 2/1, 9712 TS Groningen, The Netherlands

**Keywords:** Perceptual Gestalts, Temporal integration, Attentional blink, Rapid serial visual presentation

## Abstract

Performance in rapid serial visual presentation tasks has been shown to depend on the temporal integration of target stimuli when they are presented in direct succession. Temporal target integration produces a single, combined representation of visually compatible stimuli, which is comparatively easy to identify. It is currently unknown to what extent target compatibility affects this perceptual behavior, because it has not been studied systematically to date. In the present study, the effects of compatibility on temporal integration and attention were investigated by manipulating the Gestalt properties of target features. Of particular interest were configurations in which a global illusory shape was formed when all stimulus features were present; a Kanizsa stimulus, which was expected to have a unifying effect on the perception of the successive targets. The results showed that although the presence of a Kanizsa shape can indeed enhance temporal integration, this also was observed for other good Gestalts, such as due to common fate and closure. Identification accuracy seemed to vary, possibly as a result of masking strength, but this did not seem associated with attentional processing per se. Implications for theories of Gestalt processing and temporal integration are discussed.

It could be argued that the load on our sensory systems is increasing day by day due to technological developments. Modern means of transportation allow us to move around at high speed, while the electronic devices that we carry keep us online and in touch with others virtually continuously. Clearly, it is crucial to make the right decisions when it comes to attending to relevant objects and events, and being able to ignore those that are irrelevant—such as the incoming electronic newsletter of a clothing store while you drive.

Attention is a powerful cognitive function that allows us to make such selections. Unfortunately, it also is cognitively costly. A prime example of those costs comes from the so-called attentional blink (AB) phenomenon. The AB is the difficulty associated with identifying a second target stimulus, when it occurs in close temporal succession (200-500 ms) after a first target stimulus (Broadbent & Broadbent, 1987; Raymond, Shapiro, & Arnell, 1992; see Dux & Marois, 2009 for review). Although accounts of the AB vary, it is commonly accepted that cognitive costs are incurred to process the first target (T1), because doing so consumes limited cognitive resources, or equivalently, processing time. This then causes the attentional processing of the second target (T2) to suffer (Bowman & Wyble, 2007; Chun & Potter, 1995; Jolicœur & Dell’Acqua, 1998; Olivers & Meeter, 2008).

The AB typically has been studied in rapid serial visual presentation (RSVP) tasks, in which brief visual stimuli follow and thereby mask each other in the center of a screen. Apart from the AB, such tasks have shown that the length of the time interval that is processed as one single event by the perceptual system can have consequences for the effort needed to process the ongoing stream. This special status of perceptual events was first derived from the analysis of performance when targets in RSVP follow each other directly, at minimal stimulus onset asynchrony, without distractors in-between. In such cases, the identification of T2 often is quite good, which is called sparing, to indicate the apparent escape from the AB (for review, see Visser, Bischof, & Di Lollo, 1999). Crucially, sparing is often accompanied by a marked increase in target report order errors. This finding has prompted the idea that the two successive targets may have fallen into a single perceptual episode or event, causing temporal order information between them to be lost (Hommel & Akyürek, 2005). Temporal target integration has subsequently been implicated directly in tasks that allow not only report of individual target stimuli (e.g., “/” and “\”) but also of the temporally integrated percept of these targets (i.e., “X”), which confirmed that temporal integration drives task performance to a substantial degree at short inter-target lags (Akyürek et al., 2012; Akyürek & Wolff, 2016).

It has to be noted that alternative accounts of order reversals and sparing at Lag 1 have been put forth (Olivers, Hilkenmeier, & Scharlau, 2011; Olivers & Meeter, 2008; Wyble, Bowman, & Nieuwenstein, 2009), which propose that an attentional prior entry effect may explain both the preponderance of order errors and the comparatively high level of target identification observed at Lag 1. Although Akyürek and colleagues (2012) demonstrated that temporal integration is likely the biggest underlying factor at Lag 1, a smaller portion of trials remained in which “true” (i.e., not-integrated) order errors were observed and for which attentional effects might play a role.

Evidently, it is important to characterize the circumstances that might foster or, alternatively, prevent attentional lapses, whether they are due to short-term attentional dynamics or due to the temporal integration of targets into perceptual events. Several studies have investigated the possible effects of the stimulus properties that need to be processed on the AB. Various perceptual factors related to visual masking and target difficulty have been found to modulate AB magnitude (Chun & Potter, 1995; Giesbrecht, Bischof, & Kingstone, 2003; Seiffert & Di Lollo, 1997; Visser, 2007; although see also McLaughlin, Shore, & Klein, 2001; Ward, Duncan, & Shapiro, 1997) and to modulate target report order reversal frequency (Akyürek & Hommel, 2005), but none have considered temporal integration at Lag 1 specifically. To do so was the purpose of the present study.

For temporal integration, the visual compatibility of the successive targets is arguably paramount. At a basic level, if targets spatially overlap to a large extent, disruptive masking may result, in which the succeeding target at least partially “overwrites” the preceding one, particularly when targets are visually unfamiliar (for review, Enns & Di Lollo, 2000). Conversely, when the targets form a good figure together, their temporal integration may be facilitated. Good figures are governed by so-called Gestalt laws, which are known to exert a strong influence on perception (Wertheimer, 1938). A good figure, or Gestalt, is generated by stimulus properties such as proximity, connectedness, closure, symmetry, common fate, and continuity. Stimuli that exhibit such properties are perceptually grouped together in space (for review, Wagemans et al., 2012). Perceptual grouping is exceptionally strong for so-called Kanizsa stimuli, which induce the impression of a single emergent, illusory shape (see Fig. 1, stimuli of Experiment 2, for a classic example). Neurophysiological evidence also suggests that perceptual grouping involves processing of both actual and illusory contours, because all of these seem to take place at a relatively early processing stage (Davis & Driver, 1994) and in the same brain regions (V1/V2; Grossberg, Mingolla, & Ross, 1997; Lee, 2002; Murray, Schrater, & Kersten, 2004), although the lateral occipital complex also has been implicated in illusory contour processing (Seghier & Vuilleumier, 2006). Because Kanizsa figures thus enable the spatial integration of separate stimuli at a relatively early stage of visual processing, it seems conceivable that this may also facilitate temporal integration in RSVP.

**Fig. 1:**
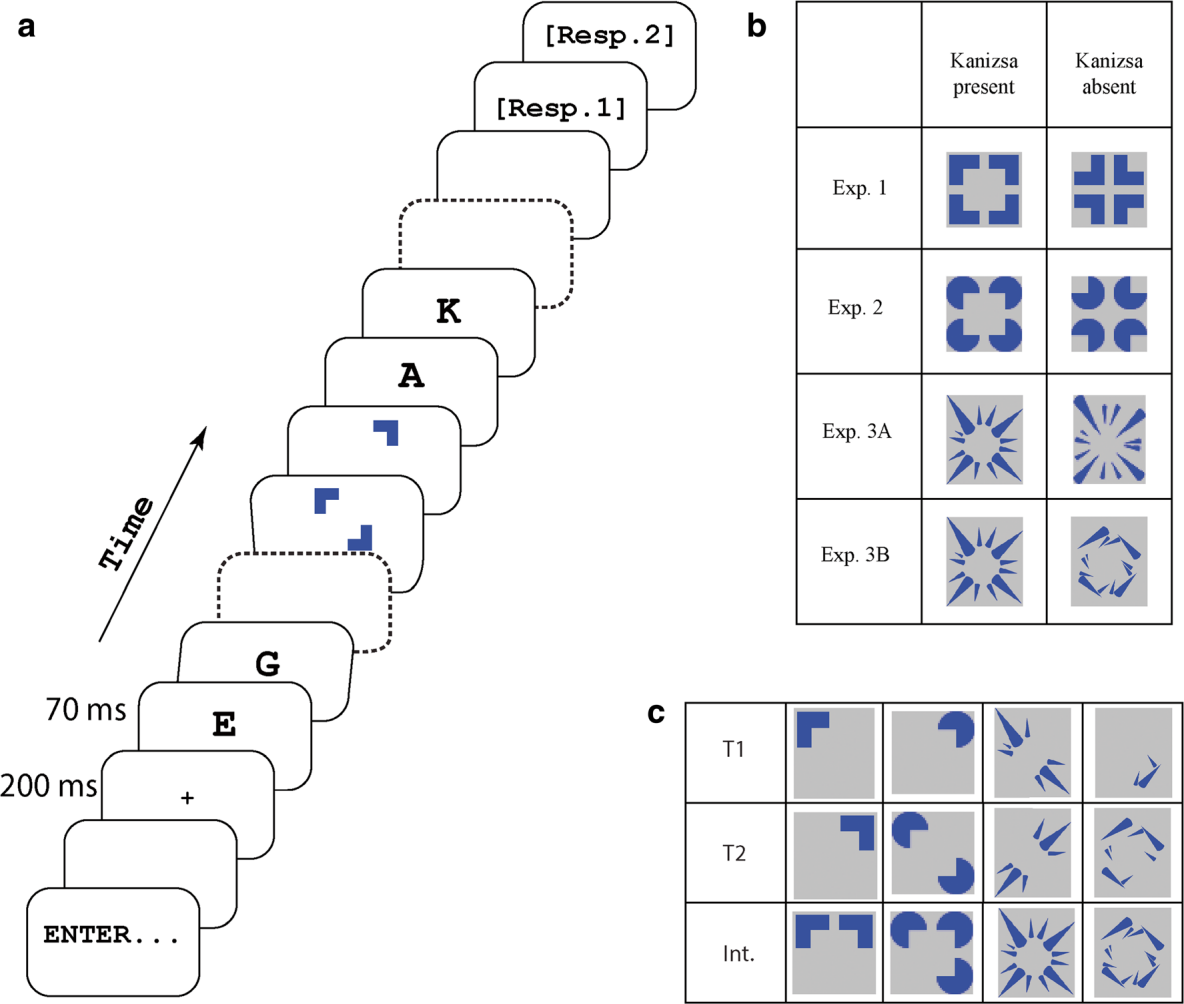
**a** Illustration of the procedure of experimental task. Letters were used as distractors, and targets appeared among these in the stimulus stream. There was a 10-ms blank interval between stimuli. Resp. refers to response prompt. **b** Target stimuli containing all four corner segments. Kanizsa-present and Kanizsa-absent columns show the experimental manipulation of the targets. On each trial, the targets contained one or more corners of these full stimuli (i.e., upper left, upper right, lower left, and lower right quadrants), without mutual overlap. **c** Examples of targets and their possible integrations. Int. is an abbreviation of integration

Similarly, because previous research has provided evidence for object-based effects on temporal attention, Kanizsa figures may affect attentional efficiency, that is, they may modulate blink magnitude at shorter lags. For instance, Kellie and Shapiro (2004; see also Raymond, 2003) showed that object file continuity decreases AB magnitude in a stimulus-morphing RSVP paradigm. When the RSVP consisted of a smooth morph of one object into another, blink magnitude was reduced compared with an RSVP in which the same images were presented in random order. The authors reasoned that a single object file (containing both targets) could be maintained in the former case, instead of having to create multiple files in the latter case. Using a multi-stream RSVP task, Conci and Müller (2009) also observed that targets in different streams that were grouped together across space by falling within the same contour region (i.e., within the same object) do not produce the same blink magnitude as targets that were not similarly grouped. This object-based interference effect was even obtained when an occluder was placed across the objects.

Based on these findings, we hypothesized that the figural goodness provided by Kanizsa figures should facilitate temporal integration and enhance or at least interact with attentional efficiency. These hypotheses were tested in a unified paradigm: As a first step, in Experiment 1, target stimuli that were used previously by Akyürek et al. (2012) were tested for possible Kanizsa effects. Subsequent experiments further examined classic Kanizsa-inducing stimulus configurations, contrasting these with configurations composed of identical elements and with varying (non-Kanizsa) Gestalt properties.

## Experiment 1

Experiment 1 was a close replication and extension of Experiment 1 reported by Akyürek and colleagues (2012). This experiment used corner segments for its target stimuli, which form a basic Kanizsa square at their center (Fig. 1). To examine the possible effect of that illusory shape, this stimulus configuration was contrasted with another in which the corners were inverted (i.e., rotated by 180°), removing the illusory square while keeping the local, low-level features of the stimuli identical.

### Method

#### Participants

Twenty-five (14 females) undergraduate students of the University of Groningen participated in the study in exchange for course credits (mean age 21.2 years, range 18-25). All participants were naïve to the purpose of the study and reported normal/corrected to normal visual acuity. The study was approved by the ethical committee of the Psychology Department of the University of Groningen (approval number 15044NE) and conducted in accordance with the Declaration of Helsinki. Written, informed consent was obtained before participation.

#### Apparatus and stimuli

Participants were seated in dimly lit sound attenuated testing cabins with a distance of approximately 60 cm from the monitor. Stimuli were presented on a 22" CRT monitor (Iiyama MA203DT). Refresh rate was set to 100 Hz with a resolution of 1280 × 768 pixels at 16-bit color depth. The study was programmed in E-prime 2.0 Professional (Psychology Software Tools) and executed in the Windows 7 operating system. A standard keyboard was used for collecting responses.

Stimuli were presented on a light gray background (RGB 192,192,192). Distractor stimuli were chosen from the full alphabet (excluding O and X), without replacement on each trial. Distractor stimuli were presented in black 52 pt Courier New Font. The fixation cross (+) was presented in the same color in 18 pt font on each trial. Target stimuli consisted of 1-4 corner segments of a square with an area of 50 by 50 pixels (1.85° by 1.85° of visual angle) in the center of the screen (Fig. 1) with the constraint that a segment was not repeated in the same trial so that there was no overlap between targets. The number of corners presented for each target was randomized, so that the total corner segments of T1 and T2 varied from two to four (e.g., one corner for T1 and another corner for T2, or one corner for T1 and two corners for T2, etc.). The length of each corner segment was 20 pixels (0.74° of visual angle) and the width was 9 pixels (0.33° of visual angle), so that the area of each corner segment was 277 pixels square. The gap between each of the corner segments was 6 pixels (0.22° of visual angle). There were two stimulus conditions; the corner segments either formed an illusory square (Kanizsa-present condition), or did not, because the segments were rotated 180° (Kanizsa-absent condition).

#### Procedure

There were 2 blocks in the experiment, each containing 216 self-paced experimental trials. Each block comprised one stimulus condition (Kanizsa-present or -absent). The order of two blocks was counterbalanced between subjects, and the trials within were randomized. The experiment started with 24 practice trials, which were omitted from analyses. Participants were offered to have a break between two blocks. The duration of the experiment was approximately 45 minutes. Participants started each trial by pressing Enter; 100 ms after pressing Enter, a fixation cross appeared on the screen for 200 ms. Then an RSVP started, accommodating 18 stimuli, each on screen for 70 ms and separated by a 10-ms blank interval. The first target appeared in the fifth or seventh position of the RSVP, which was random but equally distributed. If there was a second target, it followed the first target as the first item (Lag 1), as the third item (Lag 3), or as the eighth item (Lag 8). Seven percent of the trials consisted of only one target. Forty-six percent of the trials were dual target trials with the second target at Lag 1. Twenty-three percent of the trials consisted of dual target trials at Lag 3 and another 23% at Lag 8. Each trial was followed by two successive response prompts. These response prompts asked participants to enter T1 and T2. Participants were able to enter the two targets by pressing keys on the numeric keypad, which corresponded to the spatial locations of the corner segments (1, 2, 4, 5), followed by Enter. Moreover, participants could enter just one target by pressing the related button(s) in one of the response prompts, and only Enter in the other, or they could indicate having seen nothing by pressing Enter directly in both response prompts.

#### Design

Repeated measures analyses of variance were conducted with the design consisting of two variables: Lag (T2 lags 1, 3, 8) and Kanizsa (present when the corner segments formed an illusory square, and absent when the inversed corner segments were used). Separate analyses were conducted for T1 and T2 performance (% correct) as well as integration frequency. Unification of T1 and T2 as a single percept was defined as temporal integration. Therefore, the frequency of the exact combination of T1 and T2 as a response in one of the response prompts was calculated, with the added requirement that no response was given at the other prompt. T2 accuracy was measured in the trials on which T1 was reported correctly (T2|T1), as is commonly done. Greenhouse-Geisser corrected *p* values are reported when appropriate in all analyses. Tukey HSD tests were conducted in order to further characterize interaction effects.

### Results and discussion

**Fig. 2:**
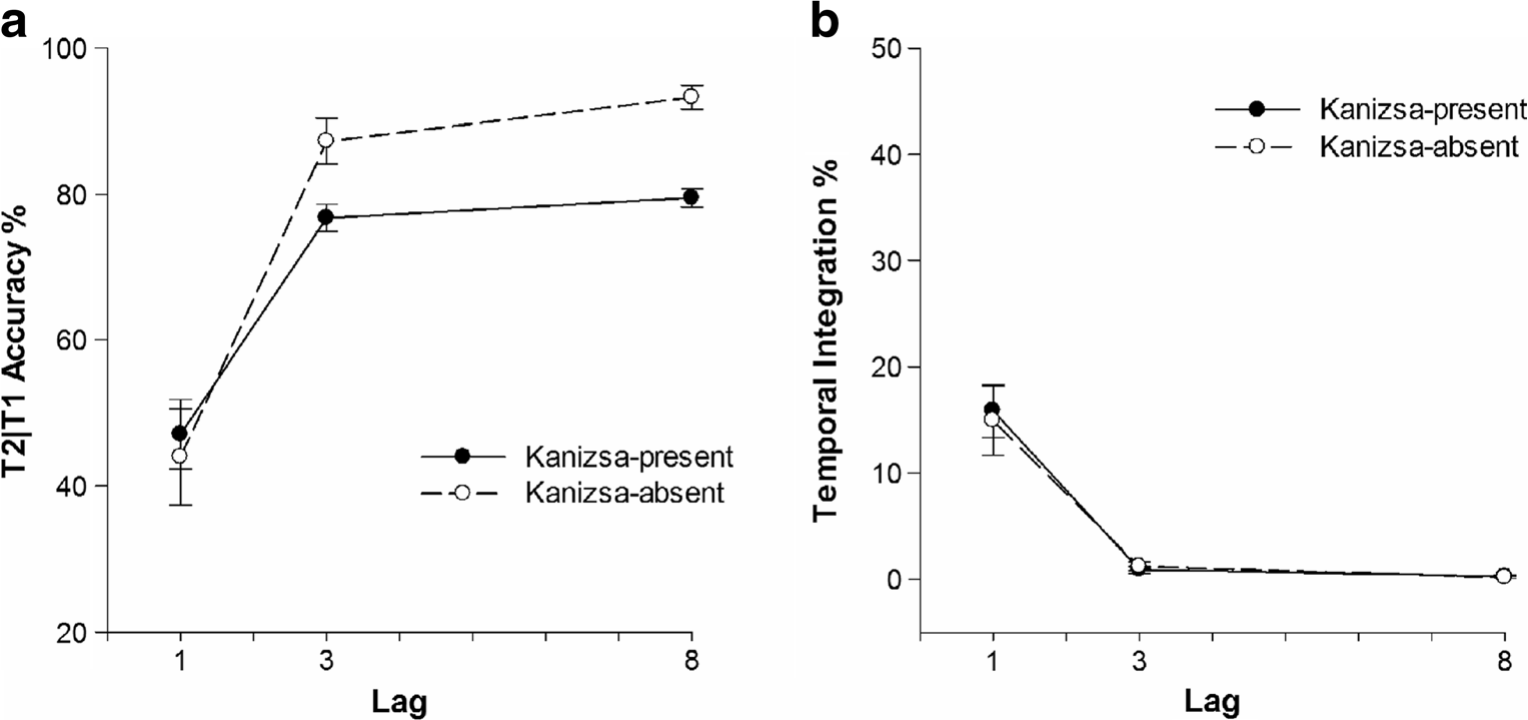
Task performance in Experiment 1 as a function of lag. Error bars represent ±SEM. **a** T2|T1 performance (T2 performance given that T1 was identified correctly in percent correct). **b** Percentage of temporal integration of T1 and T2

Participants correctly reported 85.9% (SEM = 1.5%) of one target trials, 72.8% (SEM = 0.5%) of T1 (Table 1) and 64.5% (SEM = 0.4%) of T2 in two target trials. Significant main effects of Lag and Kanizsa on T2|T1 performance existed, *F*(1, 27) = 61.03, *MSE* = 0.07, *p* < 0.01, *η* ^*2*^_*p*_ = 0.72, and *F*(1, 24) = 17.40, *MSE* = 0.01, *p* < 0.01, *η* ^*2*^_*p*_ = 0.42, respectively. T2|T1 accuracy and integration frequency are shown in Fig. 2. T2|T1 accuracy was 45.6% at Lag 1, increased to 82% at Lag 3, and further increased to 86.4% at Lag 8. T2|T1 accuracy was 67.8% when a Kanizsa contour was present and increased to 74.8% when it was not. A significant interaction effect of Kanizsa and Lag also was found on T2|T1 accuracy, *F*(1, 30) = 14.47, *MSE* = 0.01, *p* < 0.01, *η* ^*2*^_*p*_ = 0.38. Tukey HSD pairwise comparisons showed that T2|T1 accuracy on trials in which the Kanizsa was absent was significantly greater than when a Kanizsa shape was present at Lag 3 and Lag 8, but not at Lag 1, *HSD* = 8%, *p* < 0.05.

**Table 1:** Average T1 identification performance (% correct) and significant effects (indicated by asterisk symbols) observed in Experiments 1, 2, 3A, and 3B

		Lag 1		Lag 3		Lag 8		*F*	
		Mean	*SEM*	Mean	*SEM*	Mean	*SEM*	Kanizsa	Lag×Kanizsa
Exp. 1	Kanizsa-present	42.0	3.2	79.4	1.2	81.8	1.1	128.3*	0.3
	Kanizsa-absent	51.3	3.4	89.4	2.0	82.5	1.4		
Exp. 2	Kanizsa-present	37.0	3.6	92.9	1.4	95.5	1.5	6.3*	14.37*
	Kanizsa-absent	47.0	3.1	91.4	2.0	95.8	1.3		
Exp. 3A	Kanizsa-present	32.7	4.6	86.4	4.5	90.3	4.2	16.8*	2.5
	Kanizsa-absent	25.5	4.8	83.5	5.4	86.5	5.4		
Exp. 3B	Kanizsa-present	37.3	5.0	86.0	3.4	92.4	1.9	14.2*	0.9
	Kanizsa-absent	27.5	3.6	80.1	3.8	84.6	3.2

Because T1 and T2 were shown in direct succession only at Lag 1, it was expected that integration of T1 and T2 would be more frequent at Lag 1. Indeed, Lag had a significant main effect on temporal integration, *F*(1, 24) = 29.41, *MSE* = 0.03, *p* < 0.01, *η* ^*2*^_*p*_ = 0.55. Temporal integration at Lag 1 was 15.4% and decreased to 1% at Lag 3 and further decreased to 0.2% at Lag 8. Neither the main effect of Kanizsa, nor its interaction with Lag were significant.

Although there was a difference in T2|T1 accuracy at Lag 3 between the Kanizsa conditions, it seemed unrelated to attention, in view of the very similar difference observed at Lag 8, which is well outside the interval affected by the attentional blink. Thus, the results of Experiment 1 provided little evidence to suggest the presence of a Kanizsa contour might have affected the efficiency of temporal attention, nor the frequency of integration. The findings of Akyürek and colleagues (2012) should generalize across non-Gestalt stimuli.

## Experiment 2

The Kanizsa condition of Experiment 1 was intended to further scrutizine previous work (Akyürek et al., 2012), but its stimulus configuration does not strongly induce a Kanizsa shape. Thus, to test more directly whether the presence of a Kanizsa figure could principally affect temporal integration and attention, the classic Kanizsa-inducing stimulus configuration of converging “Pac-man” circles was chosen in Experiment 2 (Fig. 1).

### Method

Experiment 2 was identical to Experiment 1 with the following exceptions.

#### Participants

Twenty-five (21 females) new students participated in the study (mean age 20.36 years, range 18-26).

#### Apparatus and stimuli

Stimuli were presented on a 19" CRT monitor (Iiyama HM903DT). Stimuli were composed of (maximally) four circles with a triangular incision, known to produce a Kanizsa square when oriented appropriately (Fig. 1). The radius of the circles was 11 pixels (0.37° of visual angle) so that its area was 285 pixels square, and the distance between neighboring circles was 6 pixels (0.20° of visual angle). Similar to the procedure of Experiment 1, to implement the Kanizsa-absent condition, the stimuli were rotated 180 degrees.

### Results and discussion

**Fig. 3:**
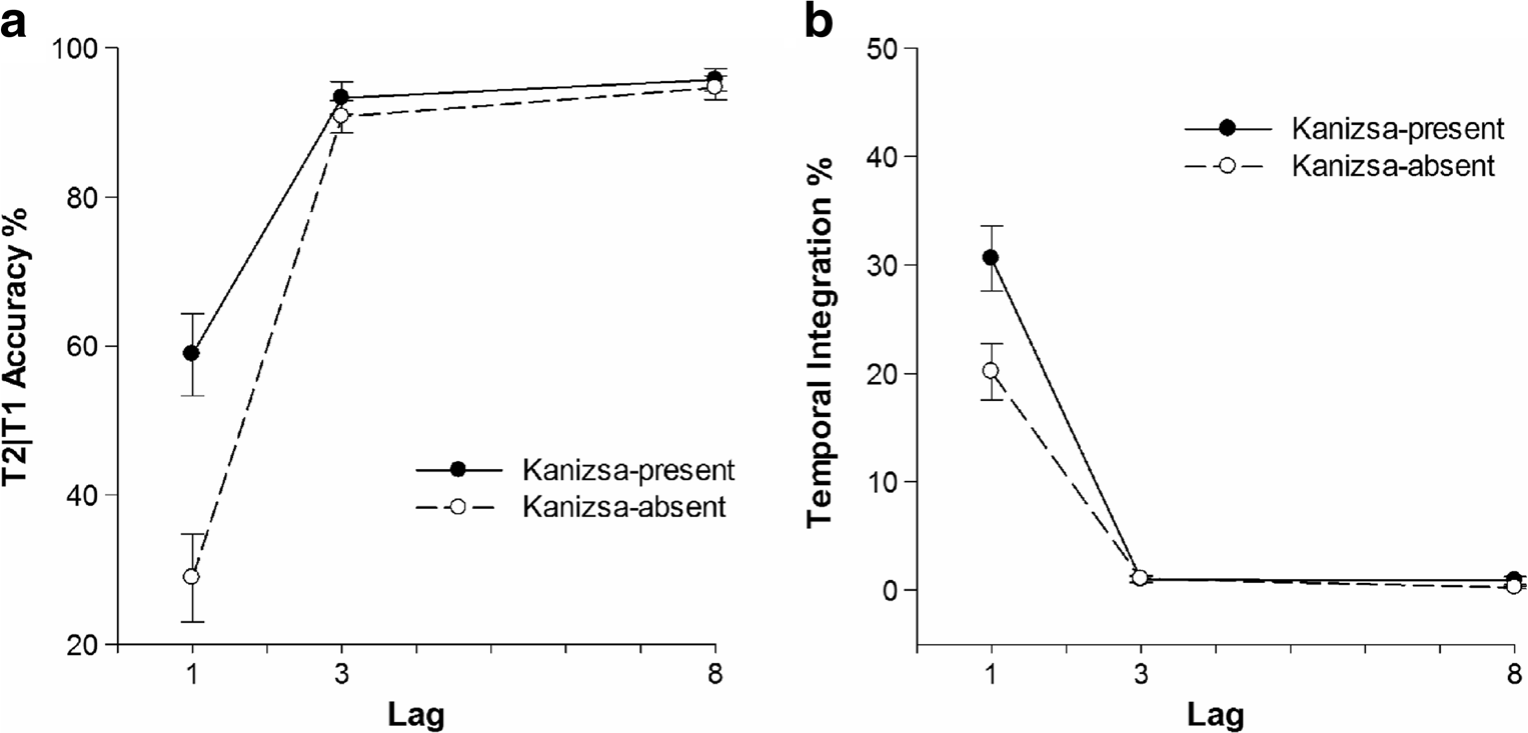
Task performance of Experiment 2 as a function of lag. Error bars represent ±SEM. **a** T2|T1 performance in percent correct. **b** Percentage of temporal integration

The overall T1 accuracy in one target trials was 91.5% (SEM = 1.3%), and in two target trials T1 accuracy was 68% (SEM = 0.5%; Table 1), and T2 accuracy was 56% (SEM = 0.5%). Similar to Experiment 1, Lag and Kanizsa had significant main effects on T2|T1 accuracy, *F*(1, 25) = 98.34, *MSE* = 0.08, *p* < 0.01, *η* ^*2*^_*p*_ = 0.80, and *F*(1, 24) = 45.09, *MSE* = 0.01, *p* < 0.01, *η* ^*2*^_*p*_ = 0.65, respectively. T2|T1 accuracy was 43.9% at Lag 1, 92% at Lag 3, and 95.2% at Lag 8. As shown in the left panel of Fig. 3, T2|T1 accuracy was 82.6% in the Kanizsa-present condition and decreased to 71.4% in the Kanizsa-absent condition, in contrast to Experiment 1. A significant interaction effect of Lag and Kanizsa on T2|T1 performance existed, *F*(1, 26) = 49.97, *MSE* = 0.01, *p* < 0.01, *η* ^*2*^_*p*_ = 0.68. Tukey HSD pairwise comparisons showed that T2|T1 accuracy at Lag 1 in the Kanizsa-present condition was significantly higher than in the Kanizsa-absent condition at lag 1, *HSD* = 9%, *p* < 0.05.

Lag and Kanizsa also had significant main effects on temporal integration, *F*(1, 24) = 95.47, *MSE* = 0.02, *p* < 0.01, *η* ^*2*^_*p*_ = 0.80, and *F*(1, 24) = 18.60, *MSE* = 0.003, *p* < 0.01, *η* ^*2*^_*p*_ = 0.437, respectively. As shown in the right panel of Fig. 3, temporal integration averaged 25.4% at Lag 1 and decreased to 1% at Lag 3 and 0.6% at Lag 8. Temporal integration in the Kanizsa-present condition was significantly higher than in the Kanizsa-absent condition. A significant interaction effect of Kanizsa and Lag was found on temporal integration as well, *F*(1, 25) = 14.59, *MSE* = 0.01, *p* < 0.01, *η* ^*2*^_*p*_ = 0.38. Pair-wise comparisons showed that temporal integration in the Kanizsa-present condition averaged 29.2% compared with 19.3% in the Kanizsa-absent condition at Lag 1, *HSD =* 8%, *p* < 0.05.

#### Between experiment comparisons

To substantiate further the effects of Kanizsa contours on T2|T1 accuracy and temporal integration frequency, two separate three-way between-subjects analyses comparing T2|T1 accuracy and temporal integration in Experiment 1 and Experiment 2 were performed. Only effects relating to differences between these experiments are reported. T2|T1 accuracy averaged 71.3% in Experiment 1 compared with 77% in Experiment 2. The interaction of Kanizsa and Experiment, as well as the interaction of Kanizsa, Lag and Experiment had significant effects on T2|T1 accuracy, *F*(1, 48) = 59.02, *MSE* = 0.01, *p* < 0.01, *η* ^*2*^_*p*_ = 0.55, and *F*(1, 58) = 5.23, *MSE* = 0.01, *p* < 0.01, *η* ^*2*^_*p*_ = 0.10, respectively. T2|T1 accuracy in the Kanizsa-present condition in Experiment 2 was 82.6% and significantly higher than the average of 67.8% observed in Experiment 1, *HSD =* 10.3%, *p* < 0.05. Post-hoc tests showed that T2|T1 accuracy in the Kanizsa-present condition of Experiment 2 was significantly greater than in Experiment 1 at each lag (1, 3, and 8). At the same time, T2|T1 accuracy in the Kanizsa-absent condition at Lag 1 in Experiment 1 averaged 44% compared with 28.9% in the same condition of Experiment 2, *HSD* = 9.2%, *p* < 0.05.

With regard to temporal integration, significant interactions of Experiment and Kanizsa, *F*(1, 48) = 8.68, *MSE* = 0.002, *p* < 0.01, *η* ^*2*^_*p*_ = 0.19, and of Experiment, Kanizsa and Lag were found, *F*(1, 50) = 7.60, *MSE* = 0.004, *p* < 0.01, *η* ^*2*^_*p*_ = 0.14. Integration frequency in the Kanizsa-present condition of Experiment 2 was significantly higher than in either Kanizsa condition of Experiment 1, *HSD =* 4.4%*, p* < 0.01. At Lag 1, temporal integration in the Kanizsa-present condition of Experiment 2 averaged 29.2% compared with 15.1% in the same condition of Experiment 1, and 14.1% in the Kanizsa-absent condition of Experiment 1, *HSD* = 5.5%, *p* < 0.05. The Kanizsa-absent condition of Experiment 2 did not reliably differ from either condition in Experiment 1 at Lag 1, averaging 19.3%.

Experiment 2 produced some notably different outcomes than Experiment 1, revealing effects of the presence of a Kanizsa figure. Both the ability to identify T2 and to integrate both targets improved at Lag 1. There also was no evidence for any effects at longer lags, which might be taken to point at an early locus in the perceptual/attentional system for the presently observed effects.

## Experiment 3A

Experiment 2 provided evidence that the presence of a Kanizsa figure facilitates temporal integration compared with a stimulus configuration in which there was no clear Gestalt. Yet unanswered is the question of whether this facilitation is exclusive to the illusory contour brought about by the Kanizsa configuration or whether other Gestalt principles would have similar effects. Experiment 3 was designed to compare the Kanizsa effect against a condition in which another good Gestalt was implemented, using the same physical features.

### Method

Experiment 3A was identical to Experiment 1 with the following exceptions.

#### Participants

Twenty-four (13 females) new students participated in the study (mean age 20.46 years, range 18-24).

#### Stimuli

In the Kanizsa condition, the stimuli were composed of cones placed around a circle, creating an illusory three-dimensional sphere, as shown in Fig. 1. In the other condition, the same cones were inverted 180°. This configuration has the properties of a good Gestalt; its features are not only similar and symmetrical but also display common fate; all the cones point to the center. Stimuli were 50 x 59 pixels (1.85° x 2.18° of visual angle) and the font of distractor stimuli was set to 60 pt. to match. The total area of the cones themselves covered 550 square pixels.

### Results and discussion

**Fig. 4:**
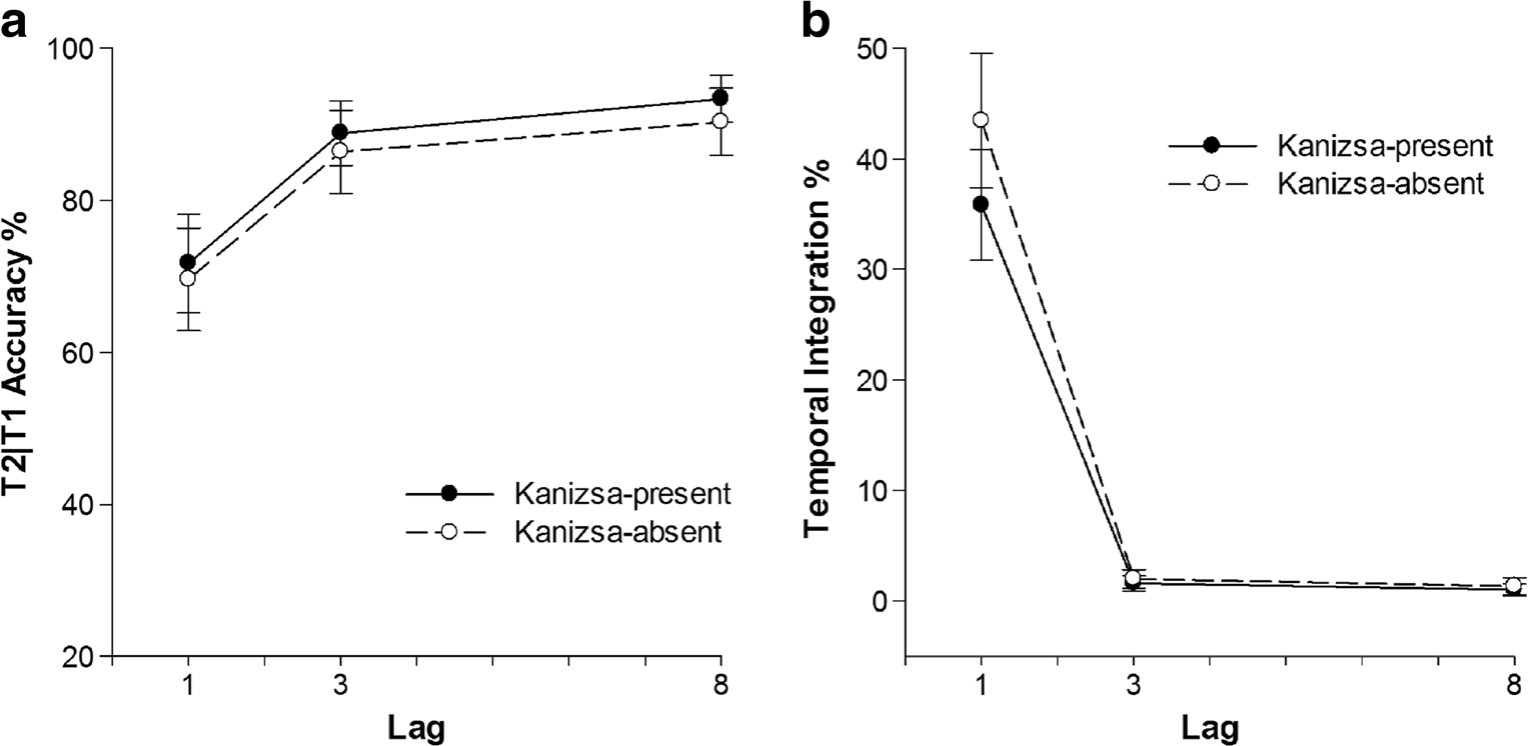
Task performance of Experiment 3A as a function of lag. Error bars represent ±SEM. **a** T2|T1 performance in percent correct. **b** Percentage of temporal integration

T1 performance in the single target condition was 81% (SEM = 1.4%), whereas T1 accuracy in the two target condition was 58% (SEM = 1%; Table 1), and T2 accuracy was 56% (SEM = 1%). Only a significant main effect of Lag on T2|T1 accuracy was found, *F*(1, 27) = 16.95, *MSE* = 0.06, *p* < 0.01, *η* ^*2*^_*p*_ = 0.42. Similar to Experiment 1 and 2, T2|T1 accuracy increased with increasing lag. T2|T1 accuracy was 70.7% at Lag 1, 87.6% at Lag 3, and 91.9% at Lag 8. Neither Kanizsa nor the interaction of Kanizsa and Lag had a significant effect on T2|T1 performance (Fig. 4 left panel).

There were significant main effects of Lag and Kanizsa on integration frequency, *F*(1, 23) = 46.91, *MSE* = 0.10, *p* < 0.01, *η* ^*2*^_*p*_ = 0.67, and *F*(1, 23) = 11.59, *MSE* = 0.002, *p* < 0.01, *η* ^*2*^_*p*_ = 0.34. Integration was most frequent at Lag 1 (Fig. 4 right panel), averaging 39.6% compared with 1.8% at Lag 3 and 1.2% at Lag 8. Contrary to expectations, temporal integration frequency in trials with the illusory Kanizsa sphere was actually slightly but significantly less than in the inverted condition. A significant interaction effect of Kanizsa and Lag was furthermore found on temporal integration, *F*(1, 24) = 9.39, *MSE* = 0.004, *p* < 0.01, *η* ^*2*^_*p*_ = 0.29. Temporal integration in the inverted condition was 43.5% at Lag 1, above the 35.8% observed in the Kanizsa sphere condition*, HSD =* 5%, *p* < 0.05.

#### Between experiment comparisons

Comparison of Experiments 2 and 3A revealed a two-way interaction of Kanizsa and Experiment, *F*(1, 47) = 10.58, *MSE* = 0.01, *p* < 0.01, *η* ^*2*^_*p*_ = 0.18, as well as a three-way interaction of Kanizsa, Experiment and Lag, *F*(2, 73) = 23.14, *MSE* = 0.01, *p* < 0.01, *η* ^*2*^_*p*_ = 0.33, on T2|T1 accuracy. T2|T1 accuracy in the Kanizsa-absent condition of Experiment 2 was 71.4% compared with 82.1% in Experiment 3A, *HSD* = 7.7%, *p* < 0.05. T2|T1 accuracy in the Kanizsa-absent condition of Experiment 3A was greater than in the Kanizsa-absent condition of Experiment 2 at Lag 1, *HSD =* 11.9%, *p* < 0.01. In addition, T2|T1 accuracy in the Kanizsa-present condition of Experiment 3A was greater than in Experiment 2 at Lag 1, *HSD* = 11.9%, *p* < 0.01.

Interaction effects of Kanizsa and Experiment as well as Kanizsa, Experiment and Lag were found on temporal integration, *F*(1, 47) = 26.31, *MSE* = 0.003, *p* < 0.01, *η* ^*2*^_*p*_ = 0.36; *F*(1, 49) = 24.10, *MSE* = 0.005, *p* < 0.01, *η* ^*2*^_*p*_ = 0.34, respectively. Overall temporal integration in the Kanizsa-absent condition in Experiment 2 was 8.8% lower than in Experiment 3A, *HSD =* 4.8%, *p* < 0.01. The combined Gestalt effects in Experiment 3A seemed stronger than in Experiment 2, and as a result both the Kanizsa-present and -absent condition of Experiment 3A caused more temporal integration at Lag 1 than they did in the Kanizsa-present condition of Experiment 2, *HSD =* 4.6%, *p* < 0.01.

In summary, the outcomes of Experiment 3A suggested that although the presence of a Kanizsa figure does result in comparatively high integration rates, nevertheless it is not special by itself. The condition in which the Kanizsa figure was not apparent, but in which a good Gestalt was present, produced as much if not more temporal integration, clearly above the levels observed in Experiment 2.

## Experiment 3B

Experiment 3B was conducted to generalize the finding of Experiment 3A that a non-Kanizsa Gestalt can be as effective as a Kanizsa figure. The motivation for conducting a further test was that in Experiment 3A the inverted, non-Kanizsa figure produced a particularly strong Gestalt, resembling an “explosion” pattern that might supersede its other properties. It is conceivable that the observed behavior resulted in part from the strength of this more subjective Gestalt. Therefore, in Experiment 3B, the cones were rotated further, so that apart from the feature similarity and symmetry present in all conditions, only the Gestalt cue of closure (marking a fairly continuous border along a rectangular center) was evident.

### Method

Experiment 3B was identical to experiment 3 with the following changes.

#### Participants

Twenty-four (10 females) new students participated in the study (mean age 21.96 years, range 19-28).

#### Stimuli

The Kanizsa condition of Experiment 3A, comprising an illusory three-dimensional sphere, was again used. In the other condition, each big cone segment in each corner was rotated 90° and small cones were rotated 135° counter-clockwise as shown in the Fig. 1. This rotation removed the Gestalt cue of common fate, thereby taking away the impression of an explosion pattern. The alignment of the cones along the edges of a rectangular center shape now introduced the Gestalt cue of closure, thereby unifying the corner segments within a single coherent figure without relying on an illusory contour.

### Results and discussion

**Fig. 5:**
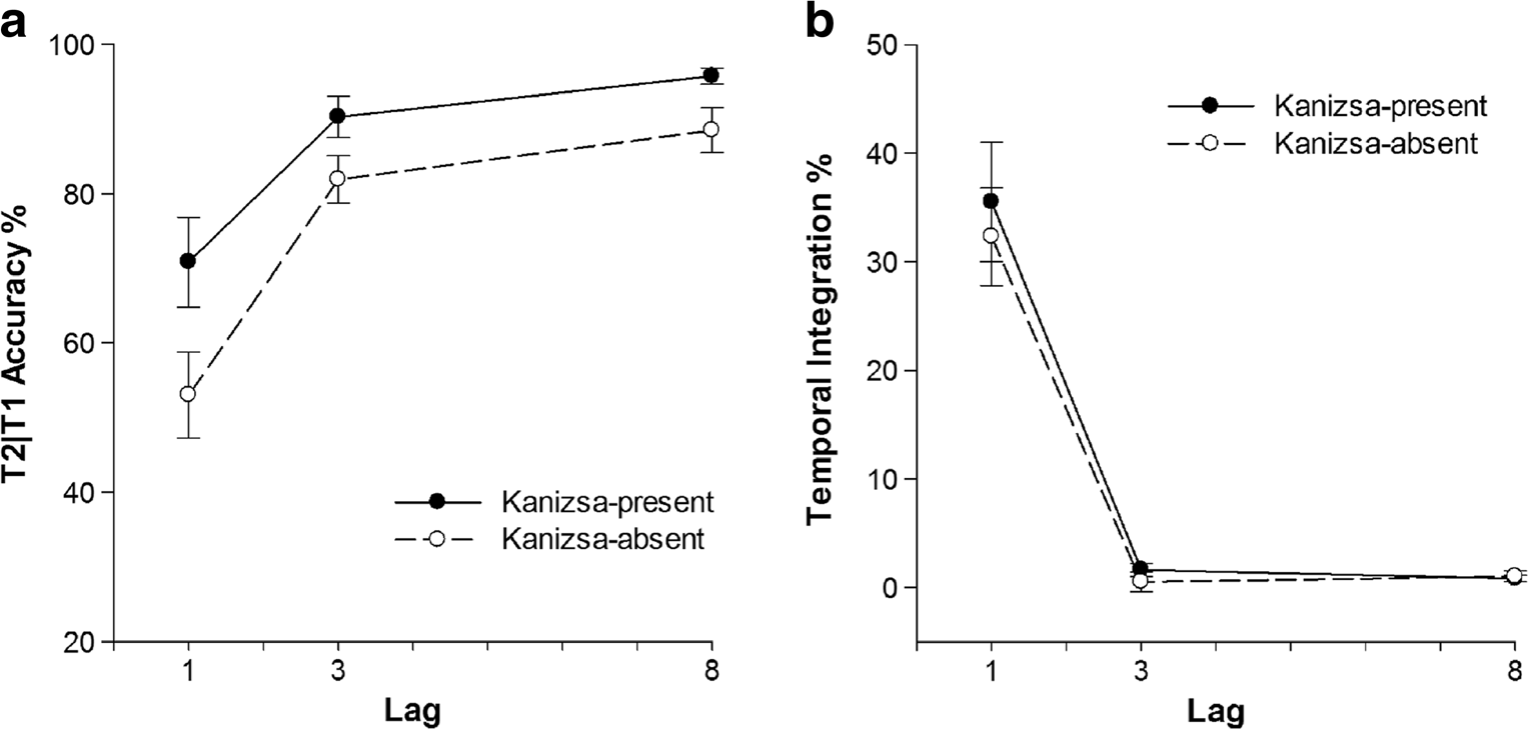
Task performance of Experiment 3B as a function of lag. Error bars represent ±SEM. **a** T2|T1 performance in percent correct. **b** Percentage of temporal integration

T1 performance averaged 83.5% (SEM = 0.5%) in the single target condition, and 59% (SEM = 0.5%) of T1 (Table 1) and 55% (SEM = 0.5%) of T2 in the two target conditions. There were significant main effects of Kanizsa and Lag on T2|T1 accuracy, *F*(1, 23) = 23.67, *MSE* = 0.02, *p* < 0.01, *η* ^*2*^_*p*_ = 0.51, and *F*(1, 28) = 34.96, *MSE* = 0.06, *p* < 0.01, *η* ^*2*^_*p*_ = 0.60. T2|T1 accuracy in the Kanizsa condition averaged 85.6%, whereas the rotated condition averaged 74.4%. T2|T1 accuracy at Lag 1 was 61.9% and increased to 86.1% at Lag 3 and to 92.1% at Lag 8 (Fig. 5 left panel).

Only Lag had a main effect on temporal integration, *F*(1, 23) = 45.17, *MSE* = 0.07, *p* < 0.01, *η* ^*2*^_*p*_ = 0.66. Temporal integration frequency at Lag 1 was 33.9% and decreased to 2% at Lag 3 and to 0.9% at Lag 8. No effects of Kanizsa were apparent (*F*’s < 2.11), confirming that with the presently used stimuli, the presence of a Kanizsa figure did not seem to further enhance target identification nor integration frequency compared with the non-Kanizsa Gestalt condition.

#### Between experiment comparisons

When comparing T2|T1 accuracy between Experiments 3A and 3B, an interaction of Kanizsa and Experiment on T2|T1 was found, *F*(1, 46) = 7.18, *MSE* = 0.02, *p* < 0.01, *η* ^*2*^_*p*_ = 0.14. T2|T1 accuracy in the Kanizsa-absent condition of Experiment 3A was higher than in the the same condition of Experiment 3B, *HSD* = 7.8%, *p* < 0.05. Thus, the weaker Gestalt in the latter experiment caused T2|T1 accuracy to decrease.

A significant interaction of Kanizsa and Experiment, *F*(1, 46) = 8.97, *MSE* = 0.002, *p* < 0.01, *η* ^*2*^_*p*_ = 0.16, and also of Kanizsa, Lag, and Experiment, *F*(1, 48) = 9.77, *MSE* = 0.002, *p* < 0.01, *η* ^*2*^_*p*_ = 0.18, was found on temporal integration frequency. Post-hoc tests showed that overall temporal integration frequency in the rotated condition of Experiment 3B was significantly lower than in the same condition of Experiment 3A, presumably as a result of the weaker Gestalt in the former experiment, *HSD* = 3.8%, *p* < 0.05. At Lag 1, the removal of the explosion pattern from the Kanizsa-absent condition in Experiment 3B induced a significant decrease of 11.2% in temporal integration frequency from the level observed in the Kanizsa-absent (explosion-present) condition in Experiment 3A, *HSD* = 4.4%, *p* < 0.01.

Between experiment comparisons of Experiment 2 and 3B revealed a three way interaction of Kanizsa, Experiment and Lag on T2|T1 accuracy, *F*(1, 58) = 5.85, *MSE* = 0.02, *p* < 0.05, *η* ^*2*^_*p*_ = 0.11. T2|T1 accuracy in either Kanizsa condition at Lag 1 in Experiment 3B was significantly higher than in Experiment 2, *HSD =* 6.5%, *p* < 0.05. At Lag 3, T2|T1 accuracy was significantly greater in Kanizsa-absent condition of Experiment 2 than the same condition of Experiment 3B.

With regard to temporal integration frequency, only an interaction effect of Kanizsa and Experiment was significant, *F*(1, 47) = 4.90, *MSE* = 0.003, *p* < 0.05, *η* ^*2*^_*p*_ = 0.09. Overall temporal integration in the Kanizsa-absent condition of Experiment 2 was 5.1% less than in the same condition of Experiment 3B, *HSD* = 2.6%, *p* < 0.05.

Experiment 3B thus continued to show relatively high T2 identification accuracy and integration rates. The specific appearance of a Kanizsa figure that unifies the corner segments of the stimuli did not seem critical; the Gestalt cue of closure was sufficient, even if the arrangement in Experiment 3A (the explosion pattern) proved to be slightly stronger still. Importantly, both configurations proved more effective than the non-Kanizsa inverted Pac-man stimuli of Experiment 2.

## General discussion

The experiments in the present study revealed that the presence of a Kanizsa figure as well as other Gestalt cues influence performance in dual-target rapid serial visual presentation tasks. These effects seemed most consistent with regard to the frequency of temporal integration at Lag 1. Target identification performance was nevertheless also affected by the appearance of the stimuli, except in Experiment 3A. These effects were obtained at various lags and seemed related to masking effects between both targets and distractors, rather than to attentional processing.

In Experiments 1 and 3B, differences in target identification accuracy between Kanizsa and non-Kanizsa conditions were observed across all lags. By contrast, the differences in Experiment 2 were restricted to Lag 1. Both patterns can be accounted for by masking, under the assumption that the target stimuli were either primarily affected by the masking strength between targets and distractor letters, or between the targets themselves. In the former case, because targets appear amidst distractors at all lags, performance differences should not be sensitive to any particular lag, as was indeed observed in Experiments 1 and 3B. In these experiments, the evidence suggested that either the Kanizsa configuration based on corner segments or the non-Kanizsa configuration based on rotated cone segments were more strongly masked by the letter distractors. In Experiment 2, the Kanizsa-absent configuration of rotated Pac-man stimuli significantly impaired identification accuracy at Lag 1, suggesting that it was caused by the close temporal proximity of the targets themselves. Because this effect was observed on both T1 and T2 accuracy, an attentional explanation, in which the AB-sensitive T2 should presumably have been affected most, seemed less tenable.

It must be acknowledged that a unifying explanation of why some stimulus shapes seemed to be more prone to distractor- or target-related masking than others is currently lacking. A possible answer may be sought in the degree to which low-level visual processing stages are involved. Wang and colleagues (2012) showed that a Kanizsa triangle emerged to awareness faster from intraocular continuous flash suppression than a rotated Kanizsa figure, suggesting that some aspects of perceptual grouping of Kanizsa figures occur in early stages of processing. These early stages may be more involved in processing the Kanizsa figures used in Experiment 2, which elicited a strong illusory figure, than in processing the figures used in Experiment 1. Consequently, the Kanizsa figures of Experiment 1 might require the involvement of later stages of processing, which implies that ensuing masking stimuli may thereby have more impact. Because the main focus of the present paper was on Kanizsa and/or Gestalt effects on integration and attention, a full account of these seemingly unrelated masking effects falls outside its scope. Future research might more systematically consider the stimulus properties that affect masking strength and individual target detection in RSVP. It may be noted that in the context of the AB proper, masking effects have proven difficult to track in previous studies (Chun & Potter, 1995; Giesbrecht, Bischof, & Kingstone, 2003; McLaughlin, Shore, & Klein, 2001; Seiffert & Di Lollo, 1997; Visser, 2007; Ward, Duncan, & Shapiro, 1997).

The lack of an attentional effect is consistent with findings in the spatial domain by Li, Cave, and Wolfe (2008). In a series of visual search experiments, they found no evidence for an attentional benefit of Kanizsa grouping. The authors concluded that such grouping might not occur early enough for attention to benefit at a later stage of processing. This interpretation is at odds with other studies, however. For instance, in line with earlier studies (Davis & Driver, 1994), Conci et al. (2009) observed “preattentive” effects of bilateral illusory contour completion on patients suffering from visual extinction. Another event-related potential study by Conci et al. (2011) showed that the earliest components (P1, N1) already reflected differential amplitude as a function of global Kanizsa shape. It thus does not seem tenable to assume that delays in perceiving illusory contours by themselves caused the present lack of an attentional effect. In the context of RSVP, however, the delay between the successive parts of the Kanizsa figure may have been sufficient. The results suggested that the targets were individually selected in all cases and that no further attentional benefits were obtained from putting the Kanizsa parts together at a later stage, such as in working memory, which has previously been shown to make use of illusory shapes (Gao et al., 2015).

In contrast to the apparent lack of attention-related effects in the current study, the Gestalt properties of the targets did produce clear modulations of temporal integration frequency. Targets with good Gestalt properties were found to be more frequently integrated when presented in direct succession at Lag 1, which was in line with expectations. At the same time, the presence of a unifying illusory Kanizsa shape was not found to have an effect over and above that afforded by other Gestalt properties.

In all experiments, the individual target features were balanced and so by definition symmetrical along both horizontal and vertical axes, as well as similar in appearance. It could be argued that a baseline Gestalt level was present throughout compared with (hypothetical) fully non-configural stimuli. For the targets in Experiment 1, which replicated previous work (Akyürek et al., 2012), an arrangement of corner segments in which a rectangular Kanizsa shape might appear was not found to deviate from an inverted arrangement that removed the illusory contour: Both conditions resulted in comparatively modest integration rates. A direct comparison to Experiment 2, in which a traditional, strong Kanizsa-inducing stimulus configuration (Pac-man circles) was used, showed that integration frequencies in Experiment 1 were similar to integration in the non-Kanizsa condition of Experiment 2. Thus, the corner segments in Experiment 1, even when oriented along a contour, did not seem to yield noticeable Gestalt benefits over other symmetrical arrangements.

The Kanizsa condition of Experiment 2 clearly induced increased integration at Lag 1, providing evidence that the spatial compatibility afforded by the illusory figure contributed to the temporal unification of the successively presented targets. However, the results of Experiments 3A and B cast doubt on the idea that the Kanizsa contour played a special role. In these experiments, an arrangement of cone segments designed to elicit an illusory Kanizsa sphere was contrasted with fully (180°) and partially rotated cones. Importantly, the rotated non-Kanizsa conditions did retain other good Gestalt properties (common fate or closure). These proved to be as effective as the Kanizsa condition, and all conditions produced integration rates comparable to the Kanizsa condition of Experiment 2. The results suggested that any of the presently tested good Gestalt properties were conducive to temporal integration. For temporal integration in RSVP, it can be concluded that perceptual grouping on the basis of illusory contours does not specifically enhance the process.

## Appendix

There were complete Kanizsa/non-Kanizsa figures when four corner segments of the stimuli were present in all four experiments. On the basis of previous findings (Nie et al., 2016), one might argue that the results of the study might be different in terms of T2|T1 accuracy and temporal integration if the comparison of Kanizsa-present and Kanizsa-absent conditions in trials that T1 and T2 form a complete figure (with 4 corner segments). A repeated measures ANOVA was adopted for testing Kanizsa effects on T2|T1 accuracy when T1 and T2 form a complete figure, and a paired sample *t*-test for temporal integration, because integrations mostly occur at lag 1. ANOVA and *t*-test results revealed an identical pattern of difference between conditions for both T2|T1 accuracy and temporal integration except for T2|T1 performance of Experiment 1. Table 2 shows ANOVA results of T2|T1 accuracy and Table 3 shows *t*-test results for temporal integration.

**Table 2:** Average T2|T1 accuracy and ANOVA results (an asterisk symbol * indicates significant *F* values) in the trials on which the combination of T1 and T2 formed a full figure (4 corner segments)

		Lag1		Lag 3		Lag 8		*F*	
		Mean (%)	*SEM*	Mean (%)	*SEM*	Mean (%)	*SEM*	Kanizsa	Kanizsa×Lag
Exp. 1	KP	55.2	6	85.0	2.7	85.8	2.0	3.2	8.5*
	KA	42.2	7.1	84.0	4.1	89.5	2.6		
Exp. 2	KP	62.8	6.4	90.7	2.9	93.4	2.2	28.2*	24.5*
	KA	25.8	6.0	88.7	2.9	91.9	2.8		
Exp. 3A	KP	65.5	8.5	85.0	5.2	88.9	4.7	0.1	0.9
	KA	69.1	8.1	85.1	5.6	85.0	5.6		
Exp. 3B	KP	69.2	7.4	87.0	4.4	94.0	1.8	17.6*	23.0*
	KA	48.0	7.2	79.3	4.5	84.4	4.6		

KP = Kanizsa-present; KA = Kanizsa-absent condition

**Table 3:** Average temporal integration and paired-sample *t*-test results at Lag 1 in the trials on which the combination T1 and T2 formed a full figure (4 corner segments)

		Lag 1		*t*	*p*
		Mean (%)	*SEM*		
Exp. 1	KP	7.6	1.9	1.4	>0.05
	KA	5.1	1.3		
Exp. 2	KP	19.3	2.2	2.3	<0.05
	KA	13.1	2.0		
Exp. 3A	KP	25.3	4.3	3.6	<0.01
	KA	36.9	5.9		
Exp. 3B	KP	29.6	5.2	0.2	>0.05
	KA	30.0	4.0		

KP = Kanizsa-present; KA = Kanizsa-absent condition

A blocked design was used in the study so that Kanizsa-present and Kanizsa-absent trials were shown in separate blocks. Starting with Kanizsa-present trials might have a learning effect that enhances temporal integration percentage in the second, Kanizsa-absent, block, or vice versa. A repeated measures ANOVA was run to investigate the main effect of block-order, the interaction of block-order and Kanizsa, and the interaction of block-order, Kanizsa, and lag. Neither the main effect of block-order nor any two-way interaction effects with Kanizsa were significant on temporal integration. A three-way interaction effect was observed in Experiment 3A; integration was more frequent in the Kanizsa-absent condition, when participants started with the Kanizsa-absent block (50% vs. 36.9%).

**Table 4:** Block-order effects across all experiments (significant results are indicated with an asterisk)

	*F*		
	Block-order	Block-order× Kanizsa	Block-order ×Kanizsa×Lag
Exp. 1	0.14	0.26	0.62
Exp. 2	0.16	0.14	0.01
Exp. 3A	0.31	3.21	7.02*
Exp. 3B	0.78	1.02	3.31
